# DFT‐Assisted Approach to Low‐Temperature Graphene Growth on Sapphire

**DOI:** 10.1002/smll.202507332

**Published:** 2025-09-18

**Authors:** Umut Kaya, Armin Sahinovic, Leon Lörcher, Carmen Nordhoff, Yasaman Jarrahi Zadeh, Tyler Lott, Germán Sciaini, Axel Lorke, Wolfgang Mertin, Rossitza Pentcheva, Gerd Bacher

**Affiliations:** ^1^ Werkstoffe der Elektrotechnik and CENIDE University of Duisburg‐Essen Bismarckstraße 81 47057 Duisburg Germany; ^2^ Department of Physics and CENIDE University of Duisburg‐Essen Lotharstraße 1 47057 Duisburg Germany; ^3^ Department of Chemistry and Waterloo Institute for Nanotechnology University of Waterloo Waterloo N2L 3G1 Canada

**Keywords:** density functional theory (DFT), graphene, plasma‐enhanced chemical vapor deposition, sapphire

## Abstract

Controlling the direct growth of 2D materials onto dielectric substrates is considered as a key requirement for integrating these ultrathin functional materials into existing technology platforms. Here, a combined experimental and theoretical approach is presented to unravel the mechanism of low‐temperature graphene growth on sapphire, a dielectric substrate widely used in the semiconductor industry. A clear dependence of the graphene growth rate on the crystal facet is found, with the highest growth rate for *a*‐plane and *ca*‐plane, and the lowest for *r*‐plane sapphire. Density functional theory calculations reveal that the coordination environment of surface oxygen ions governs carbon adsorption energetics: lower coordinated oxygen sites on the *a*‐plane markedly enhance carbon atom binding, driving nucleation and growth, while higher coordinated oxygen sites on the *r‐*plane hinder adsorption and growth. Guided by these insights, it is demonstrated that tailoring substrate termination yields controllable graphene formation at temperatures as low as 670 °C and sheet resistances down to 1.65 kΩ □^−1^. This approach may establish a universal design principle to guide low‐temperature growth of 2D materials on non‐catalytic dielectrics.

## Graphical abstract

The crystallographic orientation of sapphire strongly affects low‐temperature plasma‐enhanced chemical vapor deposition graphene growth. Experiments combined with density functional theory reveal that surface oxygen coordination governs carbon adsorption and nucleation: lower‐coordinated sites on a‐plane sapphire promote rapid growth, while higher‐coordinated sites on r‐plane slow it down. These findings provide a general strategy to optimize 2D material integration on insulating substrates.

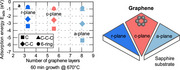

## 1 Introduction

The integration of 2D materials, such as graphene and transition metal dichalcogenides into the roadmap for next‐generation electronic and optoelectronic devices has recently been adopted by global chip manufacturers. It relies on the development of scalable, high‐quality synthesis techniques that eliminate transfer‐induced defects and contamination.^[^ ^1^, ^2^ ^]^ While exfoliated or transferred 2D materials have enabled fundamental discoveries, their practical application is constrained by challenges such as interfacial impurities, mechanical damage, and limited scalability.^[^ ^2^ ^]^ Direct growth of 2D materials on functional substrates, mainly insulators, offers a promising pathway toward wafer‐scale, transfer‐free integration of 2D materials into existing platforms. As the most prominent 2D material, graphene stands out for its exceptional electro–optical properties, including high charge carrier mobility, coupled with broadband optical transparency across the UV–vis spectrum.^[^ ^3^ ^]^

Sapphire (Al_2_O_3_) is among the most popular insulating substrates for graphene growth, owing to the templating effect of its crystalline surface, while its high dielectric constant and optical transparency further enable integration into optoelectronic devices, such as photodetectors and light‐emitting devices.^[^ ^4^ ^]^ Chemical vapor deposition (CVD) is the most commonly used method to grow graphene directly on sapphire, which can be used in a plethora of applications, for example, as transparent conducting electrodes in optoelectronics,^[^ ^5^, ^6^, ^7^, ^8^, ^9^ ^]^ or as biosensors in medical applications.^[^ ^10^, ^11^ ^]^ In other applications, a graphene interlayer is utilized for nitride epitaxy on sapphire substrates, where it acts as a buffer‐layer to reduce lattice‐mismatch‐induced defects of the nitride films.^[^ ^12^, ^13^ ^]^

Recent advances in pyrolytic CVD have enabled direct graphene growth on sapphire, overcoming its non‐catalytic nature by using high temperatures above 1000 °C, which yielded graphene with sheet resistances *R*_S_ ≈ 1 kΩ □^−1^ and a UV–vis transmittance >90%.^[^ ^5^, ^14^, ^15^, ^16^, ^17^, ^18^, ^19^, ^20^ ^]^ So far, only a few studies compare growth on various sapphire crystal planes. Saito et al. demonstrated crystal plane‐dependent pit formation at *T* > 1100 °C on *c*‐ and *a*‐plane substrates, where oxygen desorption created aluminium (Al) rich catalytic sites for graphene nucleation.^[^ ^15^ ^]^ Later, Ueda et al. showed that *r‐*plane sapphire promotes faster, higher‐quality graphene growth at *T* > 1090 °C due to enhanced catalytic activity, enabling surface protection from thermal decomposition, unlike *c‐* and *a*‐planes where growth is initiated at thermally induced pits. These studies highlight how high‐temperature CVD unavoidably alters sapphire surfaces depending on the crystal plane, leading to differing graphene growth dynamics.^[^ ^16^ ^]^

Plasma‐enhanced CVD (PECVD) has emerged to mitigate high‐temperature substrate damage. Plasma‐induced precursor decomposition enables lower growth temperatures and reduces thermal desorption effects (e.g., less pit formation). Early PECVD efforts on insulating substrates like sapphire.^[^ ^21^, ^22^, ^23^, ^24^ ^]^ yielded graphene with high defect densities (*I*_D_/*I*_G_ > 2.5),^[^ ^23^ ^]^ sheet resistances larger than 4 kΩ □^−1^,^[^ ^22^ ^]^ and poor crystallinity (*I*_2D_/*I*_G_ < 0.2).^[^ ^24^ ^]^ Recent optimizations include Zou et al.’s use of pre‐annealing (1100 °C) and chemical treatments on *a‐*plane sapphire to tailor the sapphire step‐edge morphology for graphene nanoribbon growth at 850 °C.^[^ ^25^ ^]^ Lozano et al. demonstrated that hydroxy‐group terminated *c*‐plane sapphire surfaces (vs Al‐terminated) and nitrogen carrier gas (vs hydrogen) at 800 °C reduced graphene defectiveness.^[^ ^26^ ^]^ Similarly, Muñoz et al. achieved lower sheet resistance at 700 °C on *c*‐plane sapphire using reduced gas flows and high hydrogen/carbon ratios.^[^ ^9^ ^]^ Jankauskas et al. demonstrated that the defect density is controllable over a broad range by optimizing gas flow composition and work pressure.^[^ ^27^ ^]^ Sheet resistance down to 1.8 kΩ □^−1^ was achieved, however, no graphene thickness values were stated.

Until now growth procedures have been mainly developed empirically, while an effort that directly uses first‐principles calculations to guide graphene growth on dielectric substrates is missing. Previous computational studies report partially contradicting results ranging from a weak interaction between graphene and sapphire surfaces ^[^ ^28^ ^]^ to a strong interaction at high temperatures due to trapped oxygen atoms and modifications at the graphene and sapphire interface.^[^ ^29^ ^]^ Furthermore, ab‐initio studies indicate *sp* ^3^ bonding between graphene and off‐stochiometric Al‐deficient Al_2_O_3_(0001).^[^ ^30^, ^31^ ^]^ While the formation of carbon (C)─oxygen (O) bonds and distorted C‐clusters were reported at the Al_2_O_3_(0001) surface,^[^ ^32^ ^]^ insight into the role of crystal surface facets remains unexplored.

In this work, we present a combined experimental and computational approach to elucidate and guide graphene growth on sapphire. We chose a low‐temperature PECVD approach to avoid surface reconstruction during growth and compare substrates with different surface terminations, namely *c*‐plane Al_2_O_3_(0001), *ca*‐plane Al_2_O_3_(0001 + 0.2°‐offcut toward 11–20), *a*‐plane Al_2_O_3_(11‐20), and *r*‐plane Al_2_O_3_(‐1012). High‐quality graphene was obtained on all planes for process temperatures down to 670 °C with a clear relation between growth rate and surface facet. Density functional theory (DFT) calculations including van der Walls dispersion corrections identify the most stable surface termination for each facet. The adsorption strength of individual C‐adatoms and clusters is found to be directly related to the different oxygen coordination environments at the various surface facets, providing valuable insight into the role of the substrate morphology in the initial nucleation of graphene on insulators.

## 2 Results

**Figure 1:**
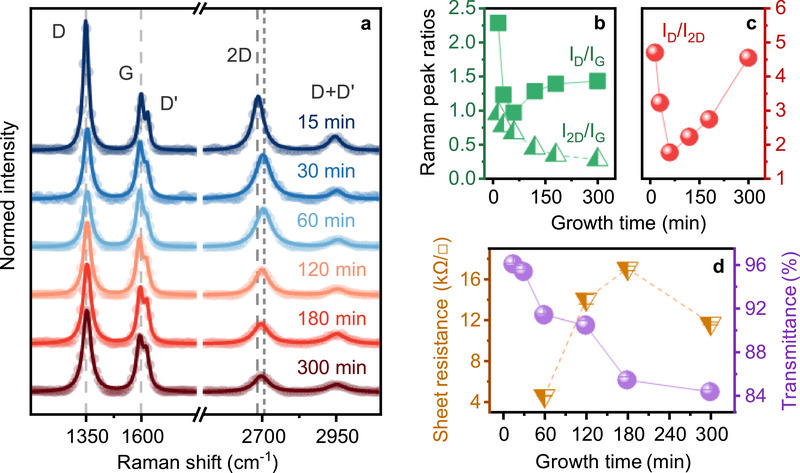
Growth time‐dependent PECVD‐growth of graphene on *ca*‐plane sapphire with constant plasma power (40 W) and growth temperature (670 °C). a) Fitted Raman spectra (lines) and raw data (dots) showing the defect related D‐peak (≈1350 cm^−1^), the sp^2^‐carbon G‐peak (1592–1600 cm^−1^) and the layer‐sensitive 2D‐peak (2694–2704 cm^−1^) at different growth times from 15 to 300 min. The grey dashed lines show a guide‐to‐the‐eye representing the shift of the 2D‐peak position *ω*_2D_ with growth time. b) Raman peak ratios *I*_D_/*I*_G_ (green squares) and *I*_2D_/*I*_G_ (white/green triangles) versus growth time. c) *I*_D_/*I*_2D_ peak ratio indicating an optimum between low defect concentration and low number of layers. d) Sheet resistance (white/orange triangles) and optical transmittance of graphene layers at 550 nm (violet dots).

As a first example, we discuss the PECVD growth of graphene on *ca*‐plane sapphire for different growth times between 15 and 300 min. Reactor geometry and process flow are shown in Figures S1,S2 (Supporting Information). In this series, plasma power and temperature were kept constant at 40 W and 670 °C, respectively. Growth time‐dependent experiments were also conducted on the other sapphire crystal planes (i.e., *c*‐, *a*‐, and *r*‐plane), which are summarized in Figure S3 (Supporting Information). **Figure** **1**a shows the fitted Raman spectra (lines) and the corresponding raw data (dots) of the graphene layers grown for different times. The typical signatures of graphene can be observed with the G‐peak and the 2D‐peak indicating the existence of an ordered sp^2^‐carbon system,^[^ ^33^ ^]^ which is further validated by a strong sp^2^ contribution in the C 1s X‐ray photoelectron spectroscopy (XPS) spectrum (Figure S4, Supporting Information). The defectivity of graphene layers is described by the D‐ (≈1350 cm^−1^), D′‐ (≈1620 cm^−1^) and D + D′‐modes (≈2940 cm^−1^), which originate from disorder‐induced phonon scattering at defects in the crystal lattice.^[^ ^33^ ^]^ PECVD graphene usually exhibits high defect‐related peaks, due to the comparably low temperatures that inhibit surface migration of adsorbed C‐atoms, which leads to high nucleation densities and hence an increased amount of grain boundaries.^[^ ^34^ ^]^

By varying the growth time, clear changes in the intensities and positions of the Raman peaks are observable (Figure 1a). The G‐peak of free‐standing, undoped, and unstrained graphene is expected to be at *ω*_G_ ≈ 1580 cm^−1^.^[^ ^33^ ^]^ We observe the G‐peak at *ω*_G_ ≈ 1600 cm^−1^ for a growth time of 15 min, shifting to ≈1592 cm^−1^ at a growth time of 300 min. This hints to p‐doped graphene,^[^ ^35^ ^]^ where the doping level decreases with increasing growth time. From literature, it is well known, that water vapor (H_2_O) in the ambient air can be the cause for the p‐doping of graphene layers.^[^ ^36^, ^37^, ^38^ ^]^ This stems from the fact that adsorbed H_2_O molecules lead to a hybridization of the electronic band structure of the graphene layers and the substrate they are suspended on.^[^ ^38^ ^]^ As our Raman measurements have been performed under atmospheric conditions, the p‐doping of the grown graphene layers by H_2_O adsorbates seems likely. Since an increase of graphene layer thickness can be expected for longer growth times, and only the uppermost of the graphene layers are being exposed to the atmospheric‐doping, an overall decrease of the p‐doping (red‐shift of *ω*_G_) for an increased growth time is conceivable.

The position of the 2D‐peak (*ω*_2D_) increases from ≈2696 cm^−1^ (15 min) to ≈2704 cm^−1^ (60 min) (see dashed grey lines in Figure 1a) and is strongly correlated to the strain in the graphene layer.^[^ ^35^ ^]^ This is a general observation, seen in all graphene layers independent of the sapphire crystal plane (Figure S5, Supporting Information). Compared to unstrained graphene (*ω*_2D_ ≈ 2680 cm^−1^), the blue‐shift in our samples indicates an enhancement of compressive strain for increasing growth times up to 60 min.^[^ ^35^ ^]^ One reason for this may originate from the low‐frequency (10 kHz) of the pulsed plasma used during PECVD growth,^[^ ^39^ ^]^ which enables H_2_^+^ ions to follow the electrical field and be accelerated toward the graphene layer, causing compressive strain.^[^ ^40^, ^41^ ^]^ Apart from this, the interaction between substrate and graphene is another frequent cause for induced strain: By simulating the interface between sapphire and graphene, Hu et al. found that the sapphire substrate is capable of shortening the C─C bonds in the graphene lattice, leading to compressive strain.^[^ ^28^ ^]^ Another reason can be found in the thermal expansion coefficient (TEC) mismatch of sapphire and graphene. At ≈700 °C, sapphire (TEC_S_ ≈ 4.77 × 10^−6 ^K^−1^)^[^ ^42^ ^]^ expands more than graphene (TEC_Gr_ ≈ −1 × 10^−6 ^K^−1^).^[^ ^43^ ^]^ During cooling, the contracting sapphire forces the bound graphene layer to compress. Hwang et al. confirmed this mechanism, showing higher growth temperatures amplify compressive strain due to increased sapphire expansion/contraction.^[^ ^44^ ^]^ To explain the increase in *ω*_2D_ (and thus the compressive strain) for longer growth times, we can assume that longer growth times result into thicker graphene layers. Bermann et al. found that thicker graphene layers bound more strongly to the substrate compared to thinner ones.^[^ ^45^ ^]^ In combination with the thermal expansion mismatch, this would lead to an increased contraction during cooling and hence higher compressive strain for thicker graphene layers.

To gain further insights into the influence of the growth time on the quality of the graphene layers, the Raman peak ratios *I*_D_/*I*_G_ (green squares) and *I*_2D_/*I*_G_ (white/green triangles) are depicted in Figure 1b. According to Ferrari et al., *I*_D_/*I*_G_ is linked to the crystallite sizes of graphene.^[^ ^46^ ^]^ Usually, *I*_D_/*I*_G_ > 1 indicates that either amorphous carbon or nanocrystalline (nc) graphene is existent. A distinction can be made by comparing *ω*_G_: For amorphous carbon *ω*_G_ ≈ 1510 cm^−1^ is expected while for nc‐graphene *ω*_G_ should be ≈1600 cm^−1^.^[^ ^33^ ^]^ Since *ω*_G_ of our PECVD‐grown graphene layers matches perfectly nc‐graphene, as can be seen in Figure 1b, the existence of amorphous carbon can be excluded. Transmission electron microscopy images confirm the nanocrystalline nature of our graphene due to a clearly visible diffraction pattern (Figure S6, Supporting Information). According to Tuinstra and Koenig, an estimate of the crystallite size *L_a_* can be extracted from *I*_D_/*I*_G_.^[^ ^46^, ^47^ ^]^ The ratio *I*_D_/*I*_G_ ≈ 2.25, as obtained for the shortest growth time (15 min), correlates with the crystallite sizes of ≈2 nm. For a growth time of 60 min, *I*_D_/*I*_G_ is reduced to ≈1, indicating crystallite sizes of *L_a_* ≈ 4 nm. For even longer growth times up to 300 min, the *I*_D_/*I*_G_ ratio and hence the overall defect density starts to increase again. Atomic force microscopy images revealed a strongly altered surface, which formed after 300 min of growth and could be one reason for the increased *I*_D_/*I*_G_ (Figure S7, Supporting Information). Generally, prolonged plasma exposure should be avoided to prevent damaging sensitive 2D‐materials over a long time for example, by ion bombardment.

The Raman peak ratio *I*_2D_/*I*_G_ plotted in Figure 1b (white/green triangles) contains information about the number of graphene layers. Generally, values of *I*_2D_/*I*_G_ > 2 suggest monolayer graphene.^[^ ^48^ ^]^ For short growth times such as 15 min, *I*_2D_/*I*_G_ ≈ 1 is observed. An increase of the growth time leads to a steady decrease down to *I*_2D_/*I*_G _≈ 0.25 at 300 min. Hence, the observed decrease in *I*_2D_/*I*_G_ with growth time hints to an increase in the number of graphene layers. In addition to the commonly used Raman peak ratios *I*_D_/*I*_G_ and *I*_2D_/*I*_G_, we have introduced the *I*_D_/*I*_2D_ ratio. Since low defect densities (i.e., low *I*_D_) and monolayers (high *I*_2D_) are desired, the *I*_D_/*I*_2D_ ratio (ideally minimal) is a convenient tool for characterizing graphene. Figure 1c displays *I*_D_/*I*_2D_ (red dots) versus growth time. For short growth times (15–30 min), *I*_D_/*I*_2D_ > 3 is observed. A minimum of *I*_D_/*I*_2D_ ≈ 1.75 can be found at a growth time of 60 min, which defines the optimal growth time for graphene on *ca‐*plane sapphire via our PECVD process. For growth times longer than 60 min, *I*_D_/*I*_2D_ starts increasing again, indicating a decreasing graphene quality.

To analyze the electrical sheet resistance *R*_S_ and the optical transmittance (*T*_opt_) of the graphene layers, four‐point probe measurements and UV–vis spectroscopy were performed. Figure 1d shows *R*_S_ (white/orange triangles) and *T*_opt_ at 550 nm (violet dots) of graphene layers grown on *ca*‐plane sapphire versus growth time. At short growth times of 15–30 min the transmittance of the graphene layers is *T*_opt_ ≈ 95.5% (15–30 min). According to Zhu et al. these values correspond to ≈1–2 layers of graphene,^[^ ^49^ ^]^ agreeing well with the Raman data. After 60 min of growth time, the transmittance indicates the existence of ≈2 additional graphene layers. Here, a sheet resistance of *R*_S_ ≈ 3.9 kΩ □^−1^ is obtained, and Hall‐effect measurements yielded mobilities of ≈45 cm^2^ Vs^−1^ and carrier densities of ≈3.4 × 10^13^ cm^−2^. Zheng et al. reported PECVD graphene mobilities of ≈16 cm^2^ Vs^−1^,^[^ ^24^ ^]^ while most studies do not provide mobility values.^[^ ^9^, ^26^, ^27^ ^]^ In contrast, thermal CVD graphene reaches mobilities >2000 cm^2^ Vs^−1^.^[^ ^14^ ^]^ The lower mobility in our case arises from the nanocrystalline nature of PECVD graphene, with grain sizes of a few nanometers and a high density of grain boundaries that hinder charge transport. For even longer growth times up to 300 min the number of graphene layers increases to ≈7, according to the transmission data.

In thermal CVD, the temperature provides the necessary energy for the precursor to decompose and the carbon species to migrate across the substrate surface to finally bond to energetically favorable sites. In PECVD, in contrast, the energy required for precursor decomposition is supplied by the plasma, enabling growth at significantly lower temperatures. The reduced growth temperature leads to decreased migration of carbon species on the surface, and changes in temperature can thus be assumed to mainly influence adsorption, nucleation, and desorption of carbon species on the sapphire substrate. These processes depend on the surface energy of the substrate, which is expected to strongly vary for different crystal planes.

**Figure 2:**
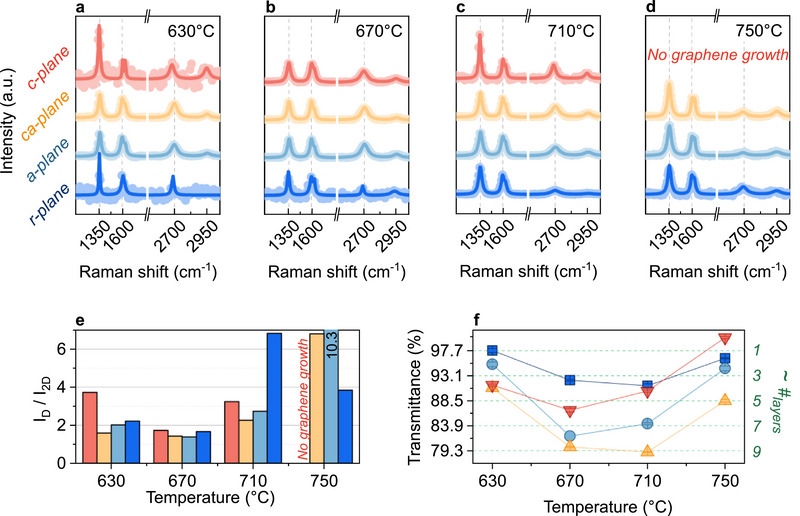
Influence of sapphire crystal orientation on graphene growth for varying temperature for *c*‐plane (red), *ca*‐plane (yellow), *a‐*plane (light‐blue), and *r‐*plane (dark‐blue) sapphire. Growth time (60 min) and plasma power (40 W) have been held constant. a–d) show the fitted Raman spectra (lines) with underlying raw data (dots) for 630, 670, 710, and 750 °C, respectively. e) *I*_D_/*I*_2D_ peak ratios derived from the Raman spectra in (a–d). f) Optical transmittance and estimated number of graphene layers at a wavelength of 550 nm versus growth temperature.

**Figure** **2** illustrates the temperature‐dependent growth of graphene on different sapphire crystal facets, comparing growth on the *c*‐plane (red), *ca*‐plane (yellow), *a*‐plane (light blue), and *r*‐plane (dark blue). This color coding is consistent throughout all further figures.

Figure 2a–d show the fitted Raman spectra (lines) with underlying raw data (dots) of graphene layers that were grown on different sapphire substrates at temperatures of 630 (a), 670 (b), 710 (c), and 750 °C (d). In addition, Figure 2e illustrates *I*_D_/*I*_2D_ for all samples. Generally, graphene grown on the *c‐*plane and *r‐*plane sapphire seems to be more defective (because of a higher *I*_D_ */I*_G_) and low Raman peak intensities (seen as low signal‐to‐noise (SN) ratios in the spectra) indicate that the coverage on these planes may be not complete. Interestingly, a minimal *I*_D_/*I*_2D_ can be observed on all crystal planes at 670 °C, indicating the temperature where the ratio of defect formation and graphene thickness is optimized (Figure 2e). At high temperatures (750 °C), an increase in defects (increasing *I*_D_ */I*_G_) can be observed on *ca*‐plane, *a*‐plane, and *r*‐plane sapphire, whereas on *c*‐plane sapphire, no graphene signals could be detected at all. This observation may be explained by desorbing carbon from the graphene lattice at high temperatures, leaving behind vacancies and leading to an increased transmittance (Figure 2f). Interestingly though, thermally induced desorption is not seen during conventional CVD processes up to growth temperatures of 1650 °C that are typically used.^[^ ^44^ ^]^

Optical transmittance measurements can be linked to the growth rate, provided that the growth time is constant. Since unstable growth occurred at temperatures of 630 °C or below (low S/N ratios), analysis focused on the temperature range above. Figure 2f compares the transmittance at 550 nm versus growth temperature for graphene grown for 60 min at a plasma power of 40 W on sapphire with different crystal facets. Strong changes in the transmission indicate temperature‐dependent growth rates, which exhibit a significant influence on the crystal plane orientation. At 670 °C, the transmittance increases progressively from *ca‐*plane toward *r*‐plane sapphire, indicating the highest growth rates on *ca‐* and *a‐*plane sapphire, followed by *c‐* and finally *r*‐plane substrates. This observed trend is persistent up until 750 °C and strongly suggests that the sapphire surface crystal structure affects graphene growth kinetics. Interestingly, in thermal CVD (*T* > 1090 °C) a different behavior is observed, where the *r‐*plane showed the highest and the *c‐* and *a‐*planes the lowest growth rates.^[^ ^16^ ^]^ Obviously, the growth mechanism in thermal CVD is fundamentally different from PECVD due to enhanced catalytic decomposition and carbon migration, which are likely not present in our low‐temperature plasma‐assisted growth approach. In addition to these temperature‐dependent effects, ion bombardment can actively influence surface processes by removing adsorbed carbon species (C, CHx), competing with nucleation. Unlike thermal CVD, PECVD does not induce temperature‐driven surface reconstructions, which are known to modify the sapphire surface energy.^[^ ^50^ ^]^ All of this likely contributes to the different facet‐selectivity observed between thermal CVD and PECVD.

**Figure 3:**
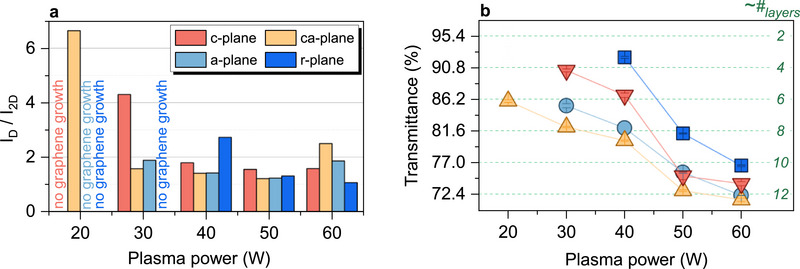
Influence of sapphire crystal orientation and plasma power on graphene growth. a) *I*_D_/*I*_2D_ peak ratios derived from the Raman spectra of graphene samples grown at plasma powers between 20 and 60 W. b) Optical transmittance and estimated number of graphene layers at a wavelength of 550 nm versus plasma power.

The plasma power is one of the inherent parameters of the PECVD process, as it controls the precursor decomposition. Higher plasma powers correlate with increased decomposition and hence exposure of the sapphire substrate to carbon species (C, CH*_x_*). Given uniform carbon exposure across sapphire substrates of varying crystal orientations, it is critical to investigate whether differences in graphene quality (≈*I*_D_/*I*_2D_) and growth rate (≈transmittance) can be observed. **Figure** **3**a shows the *I*_D_/*I*_2D_ ratios of graphene grown on *c*‐, *ca*‐, *a‐*, and *r‐*plane sapphire for differing plasma powers (for detailed data see Figure S8, Supporting Information). Starting with the lowest power of 20 W, graphene Raman signals appear only on the *ca*‐plane of sapphire. Here, the high *I*_D_/*I*_2D_ ratio (≈6.5) suggests significant defect density. Growth on the *c*‐ and *a*‐planes starts at 30 W, while successful *r‐*plane growth requires 40 W. At 50–60 W a minimal *I*_D_/*I*_2D_ ratio for all planes indicates low defect concentration and high crystallinity. This leads to a minimum sheet resistance of *R*_S _≈ 1.65 kΩ □^−1^ on *c*‐plane sapphire at 60 W. These results imply in general, that there is a threshold for the minimal plasma power required for graphene nucleation that depends on sapphire crystal orientation.

Figure 3b shows the optical transmittance at 550 nm versus plasma power for all samples where graphene growth was observed. In general, a decrease of the transmission is observed for increasing plasma power, which is expected since the plasma power is directly linked to an increased precursor decomposition and hence to the growth rate. By comparing graphene grown on differing crystal planes, significant differences in the growth rate are apparent. After growing for 60 min at 670 °C and a plasma power of 40 W, the number of estimated layers^[^ ^49^ ^]^ is ≈3 (*r*‐plane), ≈6 (*c*‐plane), ≈8 (*a*‐plane and *ca*‐plane). This resembles the trend of the growth rate observed for temperature variation (see Figure 2) and strengthens the observation that there is a significant systematic dependence of graphene growth on the sapphire crystal plane in PECVD.

**Figure 4:**
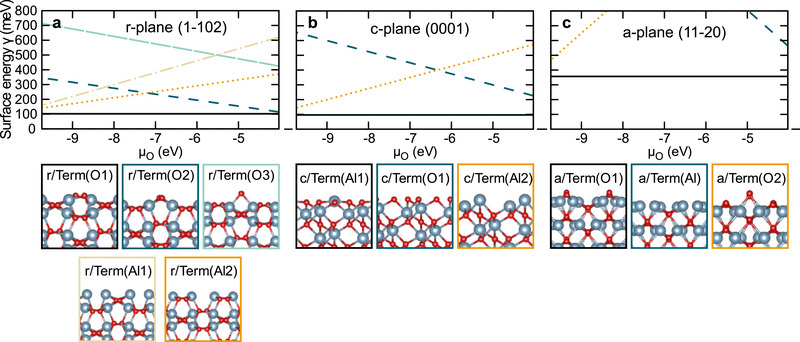
Surface phase diagram of different sapphire surface facets. The surface phase diagram shows the surface free energy of the *r*‐, *c*‐, and *a*‐plane orientation in a–c) respectively, as a function of the O chemical potential as introduced in the Experimental Section. Below a side view of the different terminations is presented and framed in different colors relating the individual termination to the lines in the phase diagram. The blue and red atoms indicate Al and O, respectively.

To shed light on the mechanism of PECVD graphene growth on sapphire, we have explored the role of the surface facets in the initial nucleation step of carbon species, using density functional theory calculations with a Becke–Johnson van der Walls dispersion correction. In a first step, we determined the most stable surface terminations of the *r‐*, *c*‐, and *a‐*plane where the growth takes place in the framework of ab initio thermodynamics^[^ ^51^ ^]^ (for further details see Experimental Section). **Figure** **4**a–c) shows the surface phase diagram for the *r*‐, *c*‐, and *a*‐crystal plane orientations as a function of the O chemical potential *µ*_O_. By truncating the surface at different planes, O or Al‐terminated surfaces were considered. The side views of the studied terminations are depicted below the surface free energy diagrams in Figure 4. The surface phase diagram indicates that the stoichiometric terminations — r/Term(O1), c/Term(Al1), and a/Term(O1) — are the most stable ones for these three surface orientations. This result is consistent with previous studies of sapphire surface terminations.^[^ ^52^, ^53^, ^54^ ^]^ Strikingly, the *a*‐plane has a three times higher surface free energy *γ* (cf. Equation (2) in Experimental Section) compared to the *c‐* and *r‐*plane, suggesting it is less stable than the latter two orientations. This correlates with the decreasing coordination of surface O: the two oxygens at the *r‐*plane orientation form either three or four O─Al bonds. At the *c‐*plane surface, oxygen has three O─Al bonds, and at the *a*‐plane, O atoms form either two or three O─Al bonds. The lower stability of the *a*‐plane with lower coordinated O sites suggests a higher reactivity.

In a second step, we search for the adsorption site with the strongest binding. A C‐atom is selectively placed above O and Al sites, and the system is fully relaxed to find the lowest energy configuration. After determining the most stable adsorption site for a single C‐atom, further C‐atoms are added consecutively to the system. Hereby, different C‐clusters are considered based on the most likely C‐particles found in the growth reactor: A single C‐atom, two and three C‐atoms forming a chain, and the less likely C_6_‐ring as a building block of graphene. Finally, by comparing the adsorption strength of the C‐clusters between the different surface facets, the most reactive surface facet for growth is determined.

**Figure 5:**
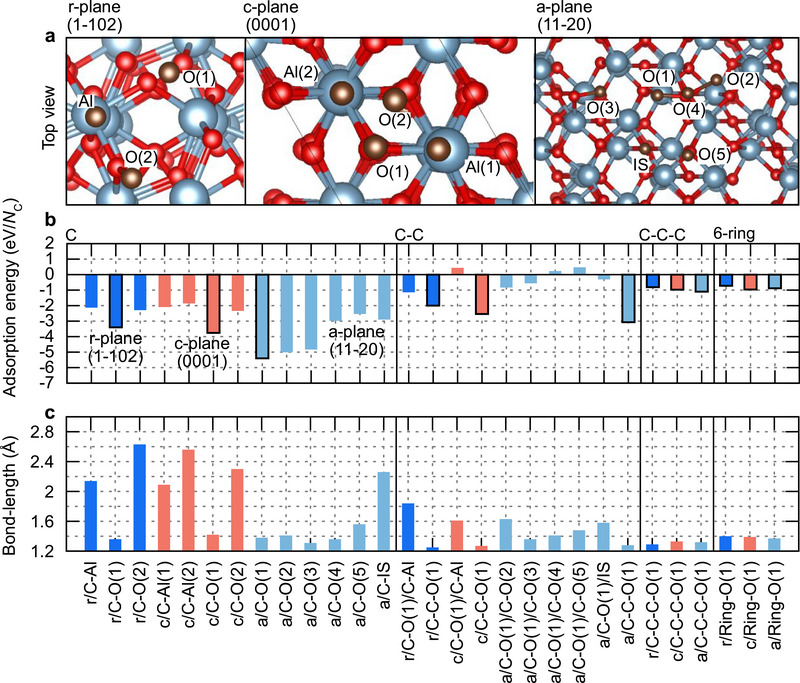
Comparison of adsorption energies of C‐clusters on *r*‐, *c*‐, and *a*‐plane sapphire facets. a) Top view of the different surface orientations with the considered adsorption sites. The brown, blue, and red spheres indicate C, Al, and O, respectively. b) Adsorption energy of single, two, three C‐atoms, and the C_6_‐ring. The dark blue, red, and light blue colors are associated with the *r*‐plane, *c*‐plane, and *a*‐plane, respectively. A black frame signals the most stable adsorption configuration. The different sites are labeled after the bonding partner of the C‐complexes and numbered if more than one option exists. c) Corresponding adsorbate‐substrate bond lengths.

The top view of the surface facets with the considered sites of the C‐clusters shown as brown spheres is displayed in **Figure** **5**a, and the corresponding adsorption energies are summarized in Figure 5b. The bond lengths are inversely correlated with the strength of the adsorption. C─Al bonds are found to be longer than 2 Å, while C─O bonds are shorter, in the range of 1.2–1.6 Å. Hence, they represent preferential sites to bind the C‐clusters. Comparing the adsorption energies after adding a second (third) C‐atom, it is evident that the formation of a bond between the two (three) C‐atoms is preferred over the adsorption of two (three) separate C‐atoms. Thus, a single adsorbed C‑atom acts as a nucleation site for other C‐atoms to bind onto. This promotes the formation of larger C‑clusters. Particularly for the *c*‐plane, individual adsorption sites could not be achieved, due to relaxation of the system leading naturally to a C─C bond. Moreover, a decrease in adsorption strength is found for larger C‐clusters showing that the single C‐atoms adsorb easier. The relaxed structures of the most stable configurations are shown in Figure S9 (Supporting Information).

A key result is the clear trend between the different crystal plane orientations which indicates a higher *a‐*plane reactivity. For the single, two, and three atom case the adsorption energy scales as |*E*_ads,a‐plane_| > |*E*_ads,c‐plane_| > |*E*_ads,r‐plane_| with the C‐atom binding to an O‐ion with two O─Al bonds (*a*‐plane), three O─Al bonds (*c*‐plane), and four Al─O bonds (*r‐*plane). This correlates with the enhanced surface free energy of the *a*‐plane shown in the surface phase diagram of all three surface facets (Figure 4). A change of the order from weakest to strongest bonding is found when the adsorption of a C_6_‐ring is considered. While the *r*‐plane still exhibits the weakest adsorption energy with −0.72 eV, the *c*‐plane allows for a much stronger bonding of the C_6_‐ring with −0.96 eV compared to the *a‐*plane with −0.88 eV.

**Figure 6:**
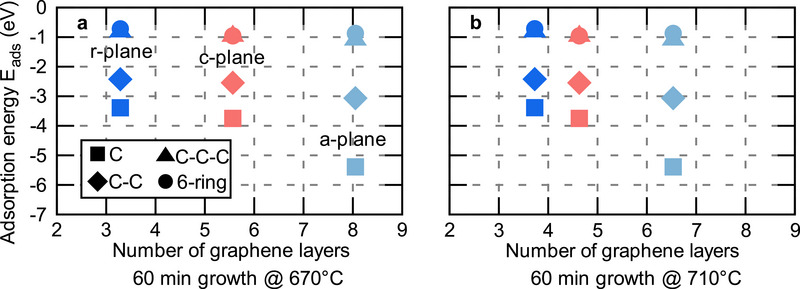
Relationship between the theoretically calculated adsorption energy of C species and the measured number of graphene layers. The theoretically calculated adsorption energy is shown against the approximate number of layers after a constant growth time of 60 min at a plasma power of 40 W for a) 670 and b) 710 °C. The different markers indicate the adsorbates considered. The colors light blue, red, and dark blue show the values for the *a*‐plane, *c*‐plane, and *r*‐plane, respectively.

**Figure** **6** shows the theoretically derived adsorption energy of the strongest binding site for each C‐cluster plotted against the approximate number of graphene layers (see Figure 2f) for different surface facets obtained from experiment. A clear relation between surface facet and number of graphene layers, that is, growth rate, is revealed. The slow growth on the *r*‐plane and the fast growth on the *a*‐plane found in experiment nicely coincides with a lower adsorption energy of C species on the *r*‐plane and a larger one on the *a*‐plane. Thus, the surface facets determine the graphene growth because of the different adsorption energies of C species, Several reports in the literature indicate a low diffusion of C‐atoms in the low‐temperature regime of PECVD growth^[^ ^55^, ^56^ ^]^ and thus carbon diffusion will play a smaller role in the PECVD growth of graphene. One can argue that the role of the substrate is largest in the initial phase of growth before substantial graphene coverage is achieved. After the initial growth of a graphene layer, the substrate effect diminishes.

We arrive at the following picture of the role of the substrate orientation in the growth: The ab‐initio results trace the different adsorption strengths back to the coordination environment of the surface terminations. A lower coordination environment of the surface O is found for the *a‐*plane compared to the *c*‐ and *r‐*plane. This leads to a higher reactivity of the *a*‐plane crystal surface. Hence, the C atoms bind stronger to the lower coordinated O ions, enabling a stronger nucleation on the *a*‐plane facet. Consequently, fast high‐quality graphene growth on insulators can be achieved if their surface is prepared to expose lower coordinated O. Hayashi et al. showed similar results for the growth of graphene on a Cu substrate, where the nucleation during graphene growth was dependent on the crystal plane of Cu, since a facet‐dependent adsorption energy of incoming carbon atoms was observed and the graphene film coverage was higher on planes with lower adsorption strength.^[^ ^45^ ^]^ Moreover, our calculations show that the initially adsorbed C‐atom acts as a nucleation center, with further incoming C‐atoms preferring to form a C─C bond over adsorption on a distant site.

## 3 Conclusion

In this study, we demonstrate that the crystallographic orientation of sapphire substrates critically governs the growth kinetics of low‐temperature graphene synthesized via PECVD. By systematically investigating *c‐*, *a*‐, *r*‐, and *ca*‐planes, we reveal facet‐dependent growth rates, with the *a*‐plane exhibiting the fastest and the *r*‐plane the slowest growth. Combining experimental observations with DFT calculations including van der Walls dispersion corrections, we trace these differences to the coordination environment of surface oxygen atoms. The *a‐*plane's lower coordinated oxygen sites enhance the adsorption strength of the carbon species, fostering rapid nucleation and growth, whereas the higher coordinated oxygen on the *r*‐plane reduces the adsorption strength. From these results, we find a high level of agreement between the experimentally observed growth rates and the simulated adsorption energetics. At the same time, it should be noted that temperature‐dependent surface kinetics such as migration and desorption processes are not captured in the simulations, as in our low‐temperature PECVD growth carbon atoms are adsorbed with a low probability of migrating along or desorbing from the surface. Essentially, our findings establish that engineering insulator surfaces with lower coordinated oxygen environments – such as the *a*‐plane of sapphire – optimizes graphene growth efficiency. This may serve as a first step toward identifying general design principles for growing 2D materials on non‐catalytic substrates.

## 4 Experimental Section

### Sapphire Wafer Preparation

Sapphire wafers with a diameter of 2‐inch and a thickness of 430 µm were used as growth substrates (Helios New Materials Limited). Four different crystallographic planes were studied in this work: *c‐*plane sapphire wafers with 0.2° off‐cut to the *m*‐plane (referred to as *c‐*plane) and to the *a*‐plane (referred to as *ca*‐plane) as well as *a*‐plane and *r*‐plane sapphire wafers. Before growth, the sapphire wafers were pre‐cut into 1 × 1 cm^2^ pieces and cleaned in boiling acetone for 3 min with a consecutive ultra‐sonication treatment for 2 min. The same procedure was repeated with boiling ethanol. Finally, the sapphire samples were rinsed with isopropanol and blown dry under a stream of nitrogen (N_2_).

### Graphene Growth via Plasma‐Enhanced Chemical Vapor Deposition

For graphene growth, the samples were loaded into a 4‐inch cold wall PECVD reactor system from AIXTRON Ltd. (Black Magic). The system consisted of a bottom heater and a graphite electrode, which was located above the bottom heater and on which the samples were placed. In a distance of 10 cm from the graphite electrode a top heater, also consisting of graphite, was located. Figure S1 (Supporting Information) shows the reactor chamber from the inside with all the mentioned components. The temperature in the system was measured with three different thermocouples located at the bottom heater, the top heater, and directly on a dummy sample, which was referred to as the surface thermocouple (SFTC), respectively. The latter was covered in a quartz sleeve to prevent damage from the plasma. The temperature measured by the SFTC was the reference for all temperatures mentioned in this work.

Methane (CH_4_), N_2_, and Argon (Ar) were used as process gases. The first process step consisted of heating up the reactor chamber with a rate of 150 °C min^−1^ in a gas flow of 800 sccm N_2_ at a pressure of 10 mbar. Once the desired growth temperature was reached, the N_2_ flux was reduced to 200, and 5 sccm of CH_4_ were introduced into the reactor. In the next step, a capacitively‐coupled direct‐current (DC) plasma was ignited between the graphite electrode at the bottom and the top heater, which acted as an electrode at the same time. The plasma was pulsed at a fixed frequency of 10 kHz. In each pulse, the bias was reversed for 1 µs to allow for the discharge of accumulated charges on the dielectric sapphire substrate. The applied bias was dependent on the plasma power, which was systematically varied in this work. Detailed information about the PECVD system could be found elsewhere.^[^ ^8^, ^39^, ^57^ ^]^ After the desired growth time was reached, the heaters were turned off and the reactor was cooled down passively under a flux of 2000 sccm Ar and 500 sccm N_2_. The cool‐down took ≈40 min to reach a temperature of 200 °C at which the samples could be taken out of the reactor safely. The complete process is schematically shown in Figure S2 (Supporting Information).

### Characterization Methods

The graphene layers were extensively characterized. For Raman spectroscopy a confocal NTEGRA Spectra system from NT‐MDT with an excitation wavelength of 532 nm, a laser spot size <0.5 µm, a spectral resolution <4 cm^−1^, a pinhole of 50 µm, and an integration time of 40 s was used. Optical transmittance was measured with a UV–vis Spectrophotometer UV‐2550 from Shimadzu in the spectral range of 300 to 800 nm. All transmittance measurements were compared to graphene‐free reference substrates for each sapphire crystal orientation. Topography information of the grown graphene layers on sapphire was obtained via the Dimension Icon atomic force microscope (AFM) from Bruker. For sheet resistance measurements, a four‐point probe system with a probe spacing of 1.27 mm from Ossila B.V. was used. The voltage‐ and current‐limits were set to 10.5 V and 200 mA, respectively. Each sample was measured four times, with a rotation of 90° after each measurement to counteract possible inhomogeneities in the graphene layer. Surface‐sensitive XPS measurements were performed with PHI 5000 VersaProbe II from Physical Electronics. Selected area electron diffraction (SAED) and transmission electron microscopy (TEM) imaging were carried out on the JEOL F200 STEM. The microscope was operated at 200 keV. SAED was recorded on a Gatan 4k × 4k pixel OneView 1095 camera at a camera length of 500 mm. Diffraction patterns were recorded with 20 frames acquired at an exposure time of 0.2 seconds each and a binning value of 1. The data was analysed using the Digital Micrograph software (Gatan) and FIJI. The diffraction rings were manually fitted and converted to determine the d‐spacing of the graphene film. Hall effect measurements in van der Pauw geometry were carried out to determine the carrier density and mobility. Ti/Au (5 nm/110 nm) contacts were deposited at the sample corners and exhibited ohmic behaviour. Measurements were conducted at room temperature under vacuum. A constant current was applied using a Keithley 2400 source meter, and the (Hall) voltage was measured using a Keithley 2000 Digital Multimeter. A perpendicular magnetic field of up to 1.2 T was applied using a Bruker B‐E 10 electromagnet.

### Density Functional Theory Calculations

The density functional theory calculations were performed with the projected augmented wave (PAW) method in the framework of the vienna ab initio simulation package^[^ ^58^, ^59^ ^]^ code, using the generalized gradient approximation for the exchange correlation functional in the parameterization of Perdew, Burke, and Ernzerhof.^[^ ^60^ ^]^ For accurate structural description and adsorption energies, the long‐range van der Waals interactions using the Grimme DFT‐D3 correction with Becke–Johnson damping were included.^[^ ^61^, ^62^ ^]^ The wave functions were expanded into plane waves up to cut‐off energies of 520 eV, and a 50 meV Gaussian smearing was added. To limit the spurious interactions between the simulated slabs, a 25 Å vacuum in the *z*‐direction was applied. The positions of all atoms were fully relaxed until the forces were below 10^−3^ eV Å^−1^. The adsorption energy *E*_ads_ was calculated using:

(1) $$\begin{array}{c} \;E_{\mathrm{ads}}=\frac{1}{N_{C}}\;\left(E_{Al_{2}O_{3}:C}\;-\;E_{Al_{2}O_{3}}\;-\;E_{C_{n}}\right) \end{array}$$

where *N_C_* is the total number of adsorbed C atoms. $E_{Al_{2}O_{3}:C}$ and $E_{Al_{2}O_{3}}$ represent the total energies of the Al_2_O_3_ slab with and without adsorbed C, respectively, while $E_{C_{n}}$ represents the energy of the respective isolated C_*n*_ cluster. Thus, a negative energy represented exothermic and energetically favored adsorption. The surface energy γ was calculated in the framework of ab initio thermodynamics,^[^ ^51^ ^]^ using

(2) $$\begin{array}{c} \gamma =\frac{1}{2A}\;\left(E_{Al_{2}O_{3}:\mathrm{surface}}\;-\;N_{\mathrm{Al}}\mu_{\mathrm{Al}}\;-\;N_{O}\mu_{O}\right) \end{array}$$

where *A* is the surface area, and the factor 2 accounts for the two surfaces in the symmetric slab model. $E_{Al_{2}O_{3}:\mathrm{surface}}$ is the total energy of the slab, while µ_Al_ and µ_O_ represent the chemical potentials of Al and O, respectively. In thermal and chemical equilibrium the chemical potentials were constrained by the bulk total energy of sapphire $E_{Al_{2}O_{3},\mathrm{bulk}}=2\mu_{\mathrm{Al}}+3\mu_{O}$.^[^ ^52^ ^]^ The O‐rich limit was defined by $\mu_{O}=\frac{1}{2}\;E_{O_{2},\mathrm{molecule}}$, whereas the Al‐rich limit corresponded to the decomposition of the substrate into metallic Al and was defined by µ_Al_  =  *E*_Al,bulk_  or $\mu_{O}=\frac{1}{3}\left(E_{Al_{2}O_{3},\;\mathrm{bulk}}-2\mu_{\mathrm{Al}}\right)$.

## Conflict of Interest

The authors declare no conflict of interest.

## Author Contributions

U.K. and A.S. contributed equally to this work. U.K. fabricated the graphene samples and performed characterization measurements with the assistance of L.L. and C.N., U.K. evaluated the gathered data of Raman, optical transmittance, and electrical sheet resistance measurements. A.S. conducted DFT calculations. U.K., W.M., G.B., A.S., and R.P. evaluated the comparison of experimental and theoretical data. T.L. and G.S. conducted and evaluated TEM measurements on graphene samples transferred by U.K., Y.J.Z. and A.L. conducted and evaluated Hall measurements. U.K. and A.S. wrote the manuscript, which was further improved by G.S., A.L., W.M., G.B., and R.P. All authors reviewed the results and approved the final version of the manuscript.

## Supporting information



Supporting Information

## Data Availability

The data that support the findings of this study are available from the corresponding author upon reasonable request.

## References

[1] A. Liu , X. Zhang , Z. Liu , Y. Li , X. Peng , X. Li , Y. Qin , C. Hu , Y. Qiu , H. Jiang , Y. Wang , Y. Li , J. Tang , J. Liu , H. Guo , T. Deng , S. Peng , H. Tian , T.‐L. Ren , Nano‐Micro Lett. 2024, 16, 119. 10.1007/s40820-023-01273-5 PMC10873265 38363512

[2] D. Neumaier , S. Pindl , M. C. Lemme , Nat. Mater. 2019, 18, 525. 31114067 10.1038/s41563-019-0359-7

[3] K. S. Novoselov , V. I. Fal′ko , L. Colombo , P. R. Gellert , M. G. Schwab , K. Kim , Nature 2012, 490, 192. 23060189 10.1038/nature11458

[4] E. R. Dobrovinskaya , L. A. Lytvynov , V. Pishchik , in Sapphire: Material, Manufacturing, Applications (Eds.: V. Pishchik , E. R. Dobrovinskaya , L. A. Lytvynov ), Springer‐Verlag US, Boston, MA, 2009, p. 55.

[5] H.‐A. Chen , W.‐C. Chen , H. Sun , C.‐C. Lin , S.‐Y. Lin , Semicond. Sci. Technol. 2018, 33, 025007.

[6] T. Journot , V. Bouchiat , B. Gayral , J. Dijon , B. Hyot , ACS Appl. Mater. Interfaces 2018, 10, 18857. 29745232 10.1021/acsami.8b01194

[7] J. Meier , H. Zhang , U. Kaya , W. Mertin , G. Bacher , Adv. Mater. 2024, 36, 2313037. 10.1002/adma.202313037 38810365

[8] J. Mischke , J. Pennings , E. Weisenseel , P. Kerger , M. Rohwerder , W. Mertin , G. Bacher , 2D Mater. 2020, 7, 35019.

[9] R. Muñoz , E. López‐Elvira , C. Munuera , F. Carrascoso , Y. Xie , O. Çakıroğlu , T. Pucher , S. Puebla , A. Castellanos‐Gomez , M. García‐Hernández , npj 2D Mater. Appl. 2023, 7, 57.

[10] D. J. Joe , J. Hwang , C. Johnson , H.‐Y. Cha , J.‐W. Lee , X. Shen , M. G. Spencer , S. Tiwari , M. Kim , J. Nanosci. Nanotechnol. 2016, 16, 144. 27398439 10.1166/jnn.2016.12042 PMC5724034

[11] S. Xu , S. Jiang , C. Zhang , W. Yue , Y. Zou , G. Wang , H. Liu , X. Zhang , M. Li , Z. Zhu , J. Wang , Appl. Surf. Sci. 2018, 427, 1114.

[12] H. Zhou , Y. Xu , X. Chen , Y. Liu , B. Cao , W.‐J. Yin , C. Wang , K. Xu , J. Alloys Compd. 2020, 844, 155870.

[13] Z. Chen , C. Xie , W. Wang , J. Zhao , B. Liu , J. Shan , X. Wang , M. Hong , L. Lin , L. Huang , X. Lin , S. Yang , X. Gao , Y. Zhang , P. Gao , K. S. Novoselov , J. Sun , Z. Liu , Sci. Adv. 2021, 7, abk0115. 10.1126/sciadv.abk0115 PMC8604399 34797705

[14] N. Mishra , S. Forti , F. Fabbri , L. Martini , C. McAleese , B. R. Conran , P. R. Whelan , A. Shivayogimath , B. S. Jessen , L. Buß , J. Falta , I. Aliaj , S. Roddaro , J. I. Flege , P. Bøggild , K. B. K. Teo , C. Coletti , Small 2019, 15, 1904906. 10.1002/smll.201904906 31668009

[15] K. Saito , T. Ogino , J. Phys. Chem. C 2014, 118, 5523.

[16] Y. Ueda , J. Yamada , T. Ono , T. Maruyama , S. Naritsuka , Appl. Phys. Lett. 2019, 115, 013103.

[17] C.‐J. Chang , P.‐C. Tsai , W.‐Y. Su , C.‐Y. Huang , P.‐T. Lee , S.‐Y. Lin , ACS Omega 2022, 7, 13128. 35474834 10.1021/acsomega.2c00554 PMC9026027

[18] J. Sitek , I. Pasternak , K. Czerniak‐Łosiewicz , M. Świniarski , P. P. Michałowski , C. McAleese , X. Wang , B. R. Conran , K. Wilczyński , M. Macha , A. Radenović , M. Zdrojek , W. Strupiński , 2D Mater. 2022, 9, 025030.

[19] Y. Kawai , T. Nakao , T. Oda , N. Ohtani , H. Hibino , Jpn. J. Appl. Phys. 2023, 62, 085503.

[20] S. Mohan , D. Kireev , S. Kutagulla , N. Ignacio , Y. Gu , H. Celio , X. Zhan , D. Akinwande , K. M. Liechti , ACS Appl. Nano Mater 2023, 6, 19018.

[21] L. Zhang , Z. Shi , Y. Wang , R. Yang , D. Shi , G. Zhang , Nano Res. 2011, 4, 315.

[22] D. Wei , Y. Lu , C. Han , T. Niu , W. Chen , A. T. S. Wee , Angew. Chem. 2013, 125, 14371. 10.1002/anie.201306086 24173776

[23] J. Sun , Y. Zhang , Z. Liu , ChemNanoMat 2016, 2, 9.

[24] S. Zheng , G. Zhong , X. Wu , L. D′Arsiè , J. Robertson , RSC Adv. 2017, 7, 33185.

[25] M. Zou , W. Liu , Y. Yu , S. Wang , B. Xu , L. Qian , T. Tong , J. Zhang , Nano Res. 2023, 16, 62.

[26] M. S. Lozano , I. Bernat‐Montoya , T. I. Angelova , A. B. Mojena , F. J. Díaz‐Fernández , M. Kovylina , A. Martínez , E. P. Cienfuegos , V. J. Gómez , Nanomaterials 2023, 13, 1952. 37446468 10.3390/nano13131952 PMC10343755

[27] Š. Jankauskas , Š. Meškinis , N. Žurauskienė , A. Guobienė , Nanomaterials 2024, 14, 1635. 39452971 10.3390/nano14201635 PMC11509920

[28] Y. Z. Hu , L. L. Luo , H. H. Shen , S. L. Hu , Z. Y. Tan , X. G. Long , Ceram. Int. 2022, 48, 12056.

[29] Z. Dou , Z. Chen , N. Li , S. Yang , Z. Yu , Y. Sun , Y. Li , B. Liu , Q. Luo , T. Ma , L. Liao , Z. Liu , P. Gao , Nat. Commun. 2019, 10, 5013. 31676774 10.1038/s41467-019-13023-6 PMC6825119

[30] D. Belotcerkovtceva , R. P. Maciel , E. Berggren , R. Maddu , T. Sarkar , Y. O. Kvashnin , D. Thonig , A. Lindblad , O. Eriksson , M. V. Kamalakar , ACS Appl. Mater. Interfaces 2022, 14, 36209. 35867345 10.1021/acsami.2c06626 PMC9376919

[31] R. P. Maciel , O. Eriksson , Y. O. Kvashnin , D. Thonig , D. Belotcerkovtceva , M. V. Kamalakar , C. S. Ong , Phys. Rev. Res. 2023, 5, 043147.

[32] J. Ryou , S. Hong , J. Phys. Soc. Jpn. 2013, 82, 114709.

[33] A. C. Ferrari , D. M. Basko , Nature Nanotech 2013, 8, 235. 10.1038/nnano.2013.46 23552117

[34] N. Li , D. Li , Z. Zhen , R. Zhang , R. Mu , Z. Xu , L. He , Mater. Today Commun. 2023, 36, 106568.

[35] J. E. Lee , G. Ahn , J. Shim , Y. S. Lee , S. Ryu , Nat. Commun. 2012, 3, 1024. 22929781 10.1038/ncomms2022

[36] C. Melios , A. Centeno , A. Zurutuza , V. Panchal , C. E. Giusca , S. Spencer , S. R. P. Silva , O. Kazakova , Carbon 2016, 103, 273.

[37] M. Bruna , A. K. Ott , M. Ijäs , D. Yoon , U. Sassi , A. C. Ferrari , ACS Nano 2014, 8, 7432. 24960180 10.1021/nn502676g

[38] T. O. Wehling , A. I. Lichtenstein , M. I. Katsnelson , Appl. Phys. Lett. 2008, 93, 202110.

[39] B. Bekdüz , U. Kaya , M. Langer , W. Mertin , G. Bacher , Sci. Rep. 2020, 10, 12938. 32737382 10.1038/s41598-020-69846-7 PMC7395096

[40] A. Kariminejad , E. Taheri‐Nassaj , M. Ghanbarian , S. A. Hassanzadeh‐Tabrizi , Mater. Des. 2016, 106, 184.

[41] A. Stoffel , A. Kovács , W. Kronast , B. Müller , J. Micromech. Microeng. 1996, 6, 1.

[42] W. M. Yim , R. J. Paff , J. Appl. Phys. 1974, 45, 1456.

[43] Z. Moradi , M. Vaezzadeh , M. Saeidi , Phys. A. 2018, 512, 981.

[44] J. Hwang , M. Kim , D. Campbell , H. A. Alsalman , J. Y. Kwak , S. Shivaraman , A. R. Woll , A. K. Singh , R. G. Hennig , S. Gorantla , M. H. Rümmeli , M. G. Spencer , ACS Nano 2013, 7, 385. 23244231 10.1021/nn305486x

[45] D. Berman , A. Erdemir , A. V. Sumant , Mater. Today 2014, 17, 31.

[46] A. C. Ferrari , J. Robertson , Phys. Rev. B 2000, 61, 14095.

[47] F. Tuinstra , J. L. Koenig , J. Chem. Phys. 1970, 53, 1126.

[48] T. van Nguyen , H. D. Le , C. van Nguyen , T. T. Tam Ngo , D. Q. Le , X. N. Nguyen , N. M. Phan , Adv. Nat. Sci: Nanosci. Nanotechnol. 2013, 4, 35012.

[49] S.‐E. Zhu , S. Yuan , G. C. A. M. Janssen , EPL 2014, 108, 17007.

[50] J. I. Hütner , A. Conti , D. Kugler , F. Mittendorfer , G. Kresse , M. Schmid , U. Diebold , J. Balajka , Science 2024, 385, 1241. 39265005 10.1126/science.adq4744

[51] K. Reuter , C. Stampf , M. Scheffler , in Handbook of Materials Modeling, (Ed.: S. Yip ), Springer Netherlands, Dordrecht, 2005, p. 149.

[52] X. G. Wang , A. Chaka , M. Scheffler , Phys. Rev. Lett. 2000, 84, 3650. 11019168 10.1103/PhysRevLett.84.3650

[53] T. Kurita , K. Uchida , A. Oshiyama , Phys. Rev. B 2010, 82, 155319.

[54] A. Abbaspour Tamijani , L. J. Augustine , J. L. Bjorklund , J. G. Catalano , S. E. Mason , Mol. Simul. 2022, 48, 247.

[55] A. J. Page , S. Saha , H.‐B. Li , S. Irle , K. Morokuma , J. Am. Chem. Soc. 2015, 137, 9281. 26148208 10.1021/jacs.5b02952

[56] C. Xiao , W. Wu , J. Chen , F. Zhang , C. Cai , G. Zhou , J. Phys. Chem. C 2025, 129, 13383.

[57] B. Bekdüz , Y. Beckmann , J. Mischke , J. Twellmann , W. Mertin , G. Bacher , Nanotechnology 2018, 29, 455603. 30156560 10.1088/1361-6528/aadd74

[58] G. Kresse , J. Furthmüller , Phys. Rev. B 1996, 54, 11169. 10.1103/physrevb.54.11169 9984901

[59] G. Kresse , J. Furthmüller , Comput. Mater. Sci. 1996, 6, 15.

[60] J. P. Perdew , K. Burke , M. Ernzerhof , Phys. Rev. Lett. 1996, 77, 3865. 10062328 10.1103/PhysRevLett.77.3865

[61] S. Grimme , J. Antony , S. Ehrlich , H. Krieg , J. Chem. Phys. 2010, 132, 154104. 20423165 10.1063/1.3382344

[62] S. Grimme , S. Ehrlich , L. Goerigk , J. Comput. Chem. 2011, 32, 1456. 21370243 10.1002/jcc.21759

