# Supplementary material for: DFT‐Assisted Approach to Low‐Temperature Graphene Growth on Sapphire
Source: Small. 2025 Sep 18;21(44):e07332. doi: 10.1002/smll.202507332 (PMC12590534; doi:10.1002/smll.202507332)
Supplement: Supplementary file 1 — Supporting Information [file SMLL-21-e07332-s001.docx]

Supplementary Information for

**DFT-Assisted Approach to Low Temperature Graphene Growth on Sapphire**

Authors:

Umut Kaya ^a^, Armin Sahinovic ^b^, Leon Lörcher ^a^, Carmen Nordhoff ^a^, Yasaman Jarrahi Zadeh ^b^, Tyler Lott ^c^, Germán Sciani ^c^, Axel Lorke ^b^, Wolfgang Mertin ^a^, Rossitza Pentcheva ^b^, Gerd Bacher ^a^

*(a) Werkstoffe der Elektrotechnik and CENIDE, Universität Duisburg-Essen, Bismarckstraße 81, 47057 Duisburg, Germany*

*(b) Fakultät für Physik, Universität Duisburg-Essen, Lotharstraße 1, 47057 Duisburg, Germany*

*(c) The Ultrafast Electron Imaging Lab, Department of Chemistry, and Waterloo Institute for Nanotechnology, University of Waterloo, Waterloo N2L 3G1, Canada*

Table of contents:

- Figure S1: Black Magic Pro 4-inch cold wall reactor from *AIXTRON SE*
- Figure S2: Schematic of graphene growth process with temperature over process time
- Figure S3: Raman spectra analysis for systematical growth time series
- Figure S4: XPS analysis of PECVD graphene grown on sapphire
- Figure S5: Correlation between 2D-peak position, strain and transmittance
- Figure S6: TEM analysis of PECVD graphene grown on sapphire
- Figure S7: AFM analysis of PECVD graphene grown on sapphire for various growth times
- Figure S8: Raman spectra analysis for systematical plasma power series
- Figure S9: DFT-calculated carbon adsorption sites on various sapphire crystal facets


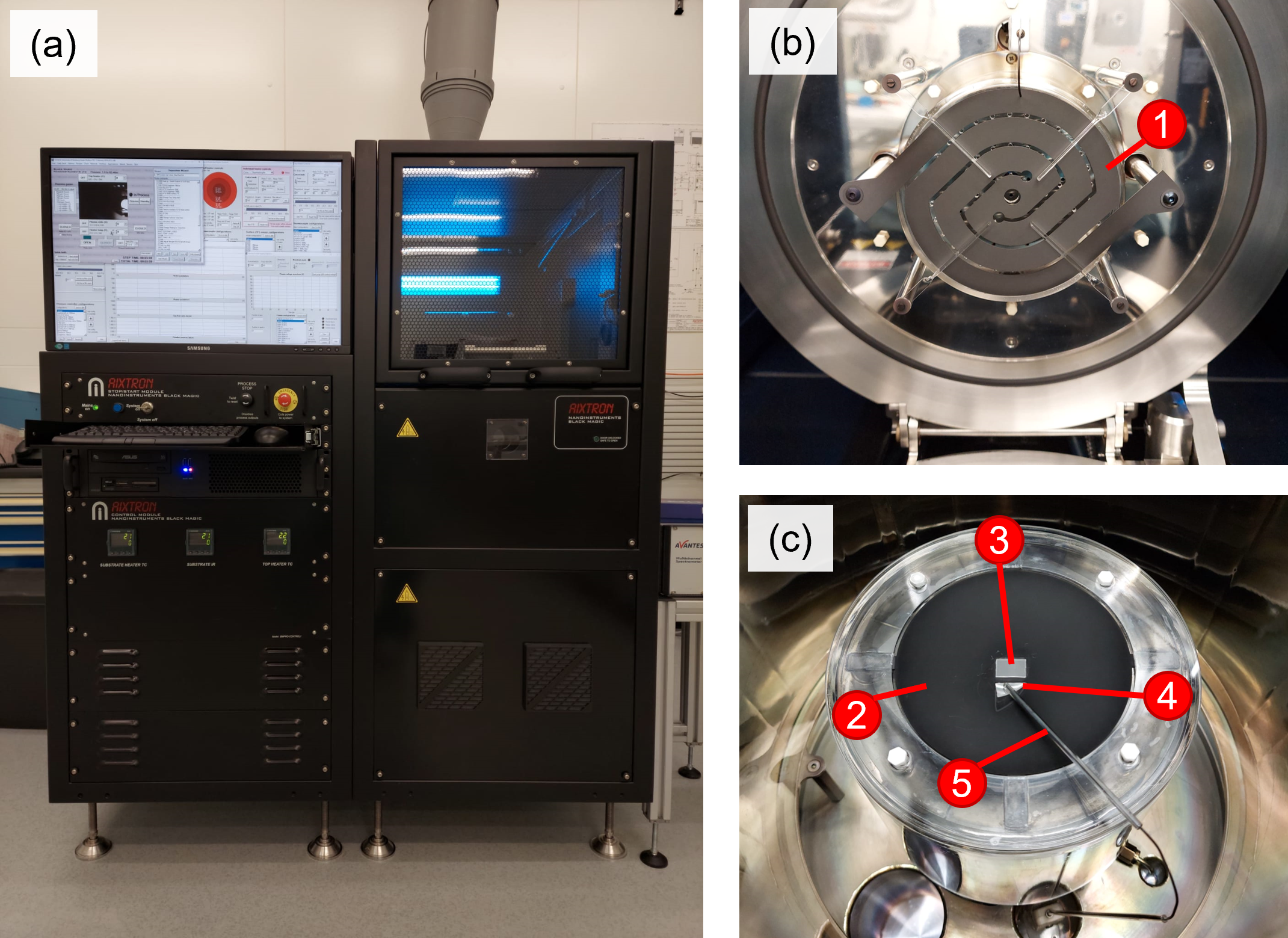


**Supplementary Figure S1 |** (a) Black Magic Pro 4-inch cold-wall CVD reactor from *AIXTRON SE*. (b) Top part of reactor chamber with top electrode/heater (1) consisting of graphite and (c) Bottom part of reactor chamber with bottom electrode (2), sample (3) and dummy wafer (4) for surface temperature measurement with the surface thermocouple (5). The bottom heater is located beneath the bottom electrode.

**Supplementary Figure S2 |** Schematic of the graphene growth process indicating temperature versus process time. The process is divided into 3 parts: (a) Heating in N_2_ atmosphere until the desired growth temperature is reached and stable. (b) Growth phase with methane introduction and plasma ignition. (c) Passive cooling phase by closing methane flow, turning-off the heaters and the methane flux and introducing Ar into the reactor chamber.

**Supplementary Figure S3 |** Raman data (solid line = fit, dots = original data) for graphene grown at different growth times (15 – 300 min) (top) and Raman peak ratios *I*_D_/*I*_G_ and *I*_2D_/*I*_G_ (bottom) on (a‑b) c‑plane, (c‑d) ca‑plane, (e‑f) a‑plane and (g‑h) r‑plane sapphire. (i) Comparison of *I*_D_/*I*_2D_ ratios for graphene grown on all studied crystal planes showing that a growth time of 60 min is optimal to minimize *I*_D_/*I*_2D_.

**Supplementary Figure S4 |** High-resolution XPS spectrum in the C1s region with sp^2^-carbon (284.8 eV), C‑O (286.3 eV) and C=O (287.7 eV) for graphene grown on ca-plane sapphire.

**Supplementary Figure S5 |** *ω*_2D_ of all graphene layers from the data presented in Figure 2a‑d versus transmittance with the same color code referring to the different crystal planes. In general, each of the planes display an increase of *ω*_2D_ for decreasing transmittance, which indicates an increasing compressive strain with additional graphene layers.


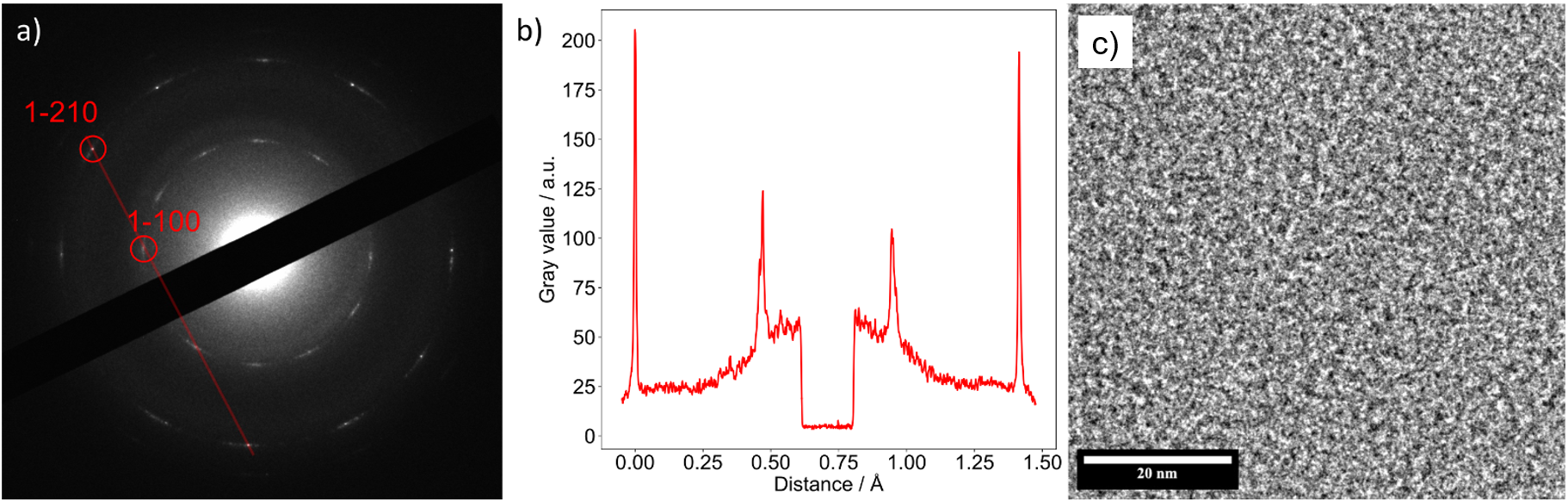


**Supplementary Figure S6 |** Selected area electron diffraction (SAED) measurements of PECVD-grown graphene on c-plane sapphire, synthesized with t = 60 min, P = 40 W, and T = 670 °C, and transferred onto a 10‑nm-thick silicon nitride window via a wet-transfer process. SAED was performed using a JEOL F200 (S)TEM operated at 200 keV. SAED was recorded on an ~ 100 nm selected area using a Gatan 4k × 4k pixel OneView camera. The diffraction image was acquired as a sum of 20 frames with an exposure time of 0.2 seconds per frame. Data analysis was carried out using Digital Micrograph (Gatan) and FIJI. **a** SAED pattern of an ~ 100 nm selected area of the graphene film, highlighting a near 12-fold symmetry with approximately 10 degrees of orientational disorder, suggesting that the growth proceeds with a certain degree of relative crystallite-to-crystallite orientational alignment. **b** Line profile analysis corresponding to the red line drawn in (a). The line passes through diffraction spots from a dominant graphene crystallite. Variations in peak intensity indicate bilayer to multilayer graphene coverage (doi:10.1038/nature05545). Using Miller–Bravais indices for a hexagonal unit cell, we assign the (1-100) and (1-210) reflections to lattice spacings of 2.13 Å and 1.23 Å, respectively (doi:10.1038/nature05545). **c** TEM micrograph of a portion of the selected area for the diffraction pattern displayed in (a). Although the image is not lattice-resolved, a moderately homogenous granular graphene film is seen to cover the imaged area.


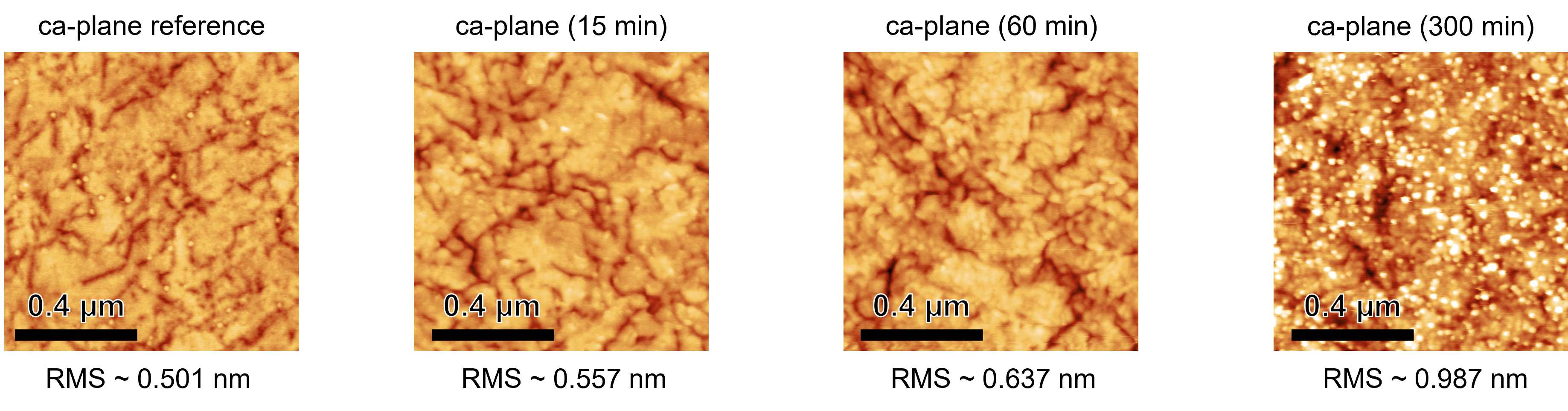


**Supplementary Figure S7 |** AFM images of a reference sapphire substrate (a) and of graphene layers grown directly on sapphire for 15 min (b), 60 min (c) and 300 min (d). The remaining process parameters were set to P = 40 W and T = 670 °C.

**Supplementary Figure S8 |** Raman data (solid line = fit, dots = original data) for graphene grown at different plasma powers (20 – 60 W) (top) and Raman peak ratios *I*_D_/*I*_G_ and *I*_2D_/*I*_G_ (bottom) on (a‑b) c‑plane, (c‑d) ca‑plane, (e‑f) a‑plane and (g‑h) r‑plane sapphire. A minimal sheet resistance of 1.65 kΩ/□ was achieved for graphene grown on c‑plane at 60 W and 670 °C for 60 min.


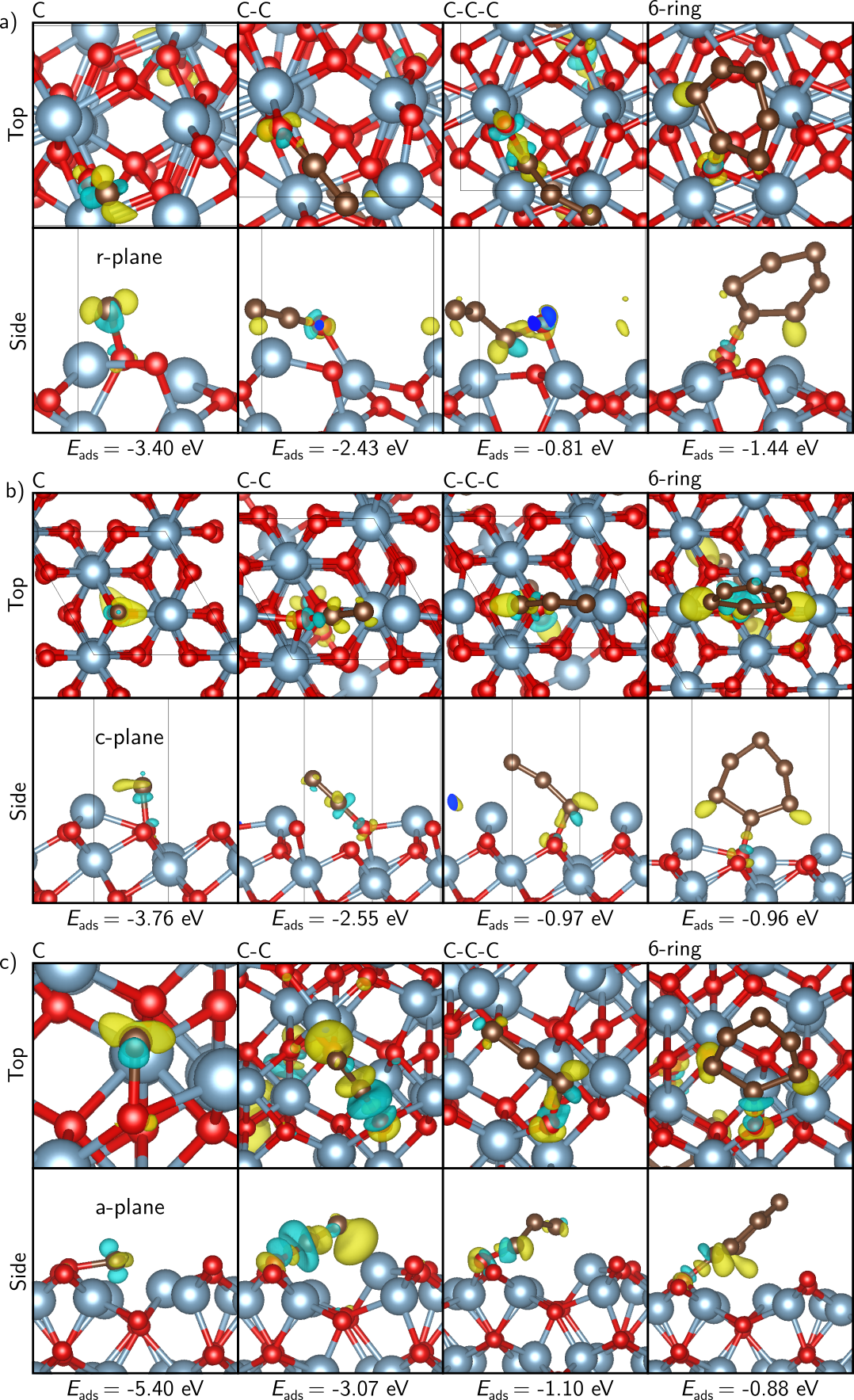


**Supplementary Figure S9 |** Adsorption sites with strongest adsorption energy for the r-/c-/a-crystal plane orientations shown in top and side view. The yellow and cyan colors represent a charge accumulation and decumulation due to bonding, respectively. a), b) and c) show the r-, c- and a-plane, respectively.
